# Dogs demonstrate perspective taking based on geometrical gaze following in a Guesser–Knower task

**DOI:** 10.1007/s10071-017-1082-x

**Published:** 2017-03-24

**Authors:** Amélie Catala, Britta Mang, Lisa Wallis, Ludwig Huber

**Affiliations:** Comparative Cognition, Messerli Research Institute, University of Veterinary Medicine Vienna, Medical University of Vienna, University of Vienna, Vienna, Austria

**Keywords:** Dog, Perspective taking, Guesser–Knower task, Geometrical gaze following

## Abstract

Currently, there is still no consensus about whether animals can ascribe mental states (Theory of Mind) to themselves and others. Showing animals can respond to cues that indicate whether another has visual access to a target or not, and that they are able to use this information as a basis for whom to rely on as an informant, is an important step forward in this direction. Domestic dogs (*Canis familiaris*) with human informants are an ideal model, because they show high sensitivity towards human eye contact, they have proven able to assess the attentional state of humans in food-stealing or food-begging contexts, and they follow human gaze behind a barrier when searching for food. With 16 dogs, we not only replicated the main results of Maginnity and Grace (Anim Cogn 17(6):1375–1392, 2014) who recently found that dogs preferred to follow the pointing of a human who witnessed a food hiding event over a human who did not (the Guesser–Knower task), but also extended this finding with a further, critical control for behaviour-reading: two informants showed identical looking behaviour, but due to their different position in the room, only one had the opportunity to see where the food was hidden by a third person. Preference for the Knower in this critical test provides solid evidence for geometrical gaze following and perspective taking in dogs.

## Introduction

Whether non-human animals are able to take the perspective of another individual or are aware of another’s knowledge state is an equally fascinating and contentious question in comparative cognition research. However, after nearly 40 years of research (Premack and Woodruff 1978), there is still neither theoretical consensus nor solid empirical evidence for this ability in non-human animals. Particularly, contentious is the question of the existence of mind-reading, mental state attribution or Theory of Mind in non-human animals, all of which describe an ability to infer the presence of mental states in others (Buckner 2014; Heyes 2015; Lurz 2011; Whiten 2013). An important and enduring difficulty is to distinguish mind-reading from cognitively simpler processes like behaviour-reading, i.e., the use of directly observable features of other animals’ situations and behaviour, and associative learning. In the recent past, attempts have been made to reduce the possibility for low-level explanations (Emery and Clayton 2016).

Following Shettleworth’s (2010) advise, sweeping anthropomorphic questions such as ‘Do animals have a Theory of Mind?’ are best answered by dissecting broad abilities into elements, some of which are phylogenetically widespread, others confined to species with specific ecologies or evolutionary histories, and some perhaps unique to humans. A highly investigated element of perspective taking and knowledge attribution is the understanding of what others can see from their perspective, especially if this deviates from the individual’s own perspective, in order to determine the others’ access to relevant information. Arguably, this requires the observer to appreciate the difference between their own and another’s line of sight (Povinelli and Eddy 1996a). For example, a subject understands that if another’s eyes are directed towards a location behind a barrier, it must alter its own position in order to see the object of its interest. This ability has been called ‘geometrical gaze following’ (Tomasello et al. 1999).

A species considered as especially skilled in responding to human communicative cues, such as pointing and gazing, is the domestic dog (recent reviews in Bensky et al. 2013; Huber 2016; Kaminski and Marshall-Pescini 2014; Wynne 2016). Indeed, dogs do not only follow the gaze of humans into distant space (Wallis et al. 2015), but at least some dogs (about a third of the tested sample) are capable of following the gaze of humans around a barrier in a food searching context (Met et al. 2014). Interestingly, when the dogs had not been primed to forage before the test, they did not follow the experimenter’s gaze behind a barrier (similarly to an earlier study by Agnetta et al. (2000) who also found negative results for gaze following in a non-foraging context).

It is still not clear what cognitive mechanisms support the ability of dogs to follow human gaze and to respond adaptively to human attentional states. In particular, the question of whether dogs understand if humans have visual access to food, or if they simply respond, because of a special sensitivity or as a result of associative learning, to perceptual cues, like seeing the human’s body or parts of it (Bräuer et al. 2004; Kaminski et al. 2009), remains to be answered. Can dogs infer from indirect cues what humans can or cannot see? Two recent studies, one framed in a non-cooperative and the other in a cooperative setting, came very close to answering these tricky questions. The first revealed that the level of illumination around the food affected whether dogs attempted to steal the food in the presence of a human (Kaminski et al. 2013). Results suggested dogs understand when the food (and therefore the area around it) is illuminated, the human can see them approaching and stealing the food.

That dogs may actually understand something about a human’s perspective has been demonstrated in the second study by using the famous ‘Guesser–Knower task’ (Povinelli et al. 1990). Maginnity and Grace (2014) showed that dogs’ choices between two human informants were not only influenced by cues related to food handling (Experiment 1), but also by cues related to the humans’ visual access to the food. In Experiment 2, dogs followed a human’s (the Knower) point to a food container after watching the food hiding (where she covered her cheeks with her hands), rather than another human’s (the Guesser) point to a different food container (after she covered her eyes with her hands during the food hiding). In Experiment 3, dogs avoided a human (the Guesser) who looked at the ceiling during the hiding of the food, and again followed the human (the Knower) who observed the hiding. Controls in two further experiments ruled out responding on the basis of unintentional cues provided by the owners, or informants, or olfactory cues. This study confirmed that dogs have a remarkable sensitivity to cues relating to humans’ attentional state, in this case the location of the experimenter’s hands on their faces, and their gaze directions.

It is still an open question if dogs can use geometrical gaze following as a perspective-taking mechanism, to assess what a human can see and therefore know. Dogs do follow the gaze of humans into distant space (Wallis et al. 2015), but this orientation response may be based on a relatively simple mechanism, to align their view with that of another individual gazing towards something (Povinelli and Eddy 1996a). This would only allow them to search for something of interest to themselves. Such an egocentric perspective was shown in another stealing task, which required dogs to infer that a human could see them, although they could not see the human. However, results showed dogs could not conceal their act of stealing from the human in the visual domain by hiding their approach when they could not see a human present. Still, they could do so in the auditory domain by preferring a silent approach to forbidden food (Bräuer et al. 2013). Although dogs seem to understand how barriers impair others’ perception (Bräuer et al. 2006), they have so far not been tested formally for geometrical gaze following.

The aim of the present study was twofold. First, on the basis of the contentious issue of perspective taking in non-human animals, especially when using the Guesser–Knower paradigm, and because most dog studies are underpowered (Arden et al. 2016), we aimed at replicating the study of Maginnity and Grace (2014). Secondly, in order to check whether the dogs’ assessment of a human’s knowledge can go beyond directly observable differences between the two informants, we conducted a variant of the Guesser–Knower task in which both human informants behaved identically: they both looked in the same direction but differed in whether they could see the baiting process. Importantly, the object of interest to the human was not visible to the dogs; therefore, they could not simply use the eye-object line (Heyes 1994; Udell and Wynne 2011), but must infer from the humans’ gaze direction what they can see or not, i.e., geometrical gaze following. In order to prevent the dogs from using unintentional cues, like pointing more confidently, to discriminate between the informants, the experimenters exchanged roles (Knower vs Guesser) repeatedly in each test, and both were always informed about the food location. Finally, to rule out associative learning, we analysed the first-trial data and checked for possible changes in the dogs’ performance across trials.

## Methods

### Subjects

As in Maginnity and Grace (2014), 16 privately owned dogs (eight males, eight females; mean age = 4.8 years; various breeds) participated in this study. All subjects lived as pet dogs with their owners, who volunteered to bring their dogs to the Clever Dog Lab for this study. These dogs were naïve to any experiment involving perspective taking, but some of them have participated in other types of experiments at the Clever Dog Lab before, with only five in a pointing task (see Table 1).

**Table 1:** Individual characteristics (sex, age, breed) of the subjects, their pre-experimental experience, the percentages of Knower choices and the first-trial performances in the three tests

Dog	Sex	Age	Breed	Experience^a^	GP			GA			GLA		
					Mean	First trial	NVT^b^	Mean	First trial	NVT	Mean	First trial	NVT
Clio	F	1.5	Mix	TS, II	0.58	K	24	**0**.**88**	K	24	**0**.**83**	K	24
Lola	F	3	Mix Shepherd	P, TS, II, Ps	0.61	K	23	0.62	G	21	0.63	K	24
Freyja	F	2.5	Czechoslovakian Wolfdog		0.52	G	23	**0**.**88**	K	24	0.58	G	24
Louise	F	9	Mix		0.63	G	24	**0**.**78**	K	23	0.63	K	24
Hybie	F	7	Labrador Retriever	ET, TS, II, Ps	0.64	K	22	**0**.**91**	G	22	0.54	K	24
Tuukka	F	2	Mix	P, ET, TS, II, Ps	0.57	G	23	**0**.**71**	K	24	0.67	K	24
Haly	F	5	Jack Russell Terrier		0.50	K	24	0.67	K	24	**0**.**71**	K	24
Izy	F	4	Podenco		0.41	K	22	**0**.**71**	K	24	0.65	K	23
Mowgli	M	3	Mix	II	0.58	K	24	0.67	G	24	0.67	K	24
Benji	M	6	Mix	P, ET, II, Ps	0.50	K	24	0.65	K	23	0.63	K	24
Koda	M	4.5	Mix German Shepherd		0.42	G	24	**0**.**87**	G	23	0.58	K	24
Cameron	M	3.5	Border Collie	ET, II, Ps	**0**.**79**	K	24	**0**.**83**	–	23	0.58	K	24
Bucksi	M	7	Papillon		**0**.**27**	G	22	0.65	K	21	**0**.**71**	K	24
Patrasch	M	8.5	Mix Spitz	TS	0.63	G	24	0.54	K	24	0.43	K	23
Charlie	M	7	Bearded Collie	P, II, Ps	0.67	K	24	0.67	K	24	0.58	G	24
Cookie	M	4	Bearded Collie	P, II	0.70	K	23	0.54	K	24	0.46	G	24

Bold typeface indicates performance significantly different from chance

*GP* Guesser Present, *GA* Guesser Absent, *GLA* Guesser Looking Away

^a^Experience: *TS* Touch screen, *II* Inhibition control; Inequity aversion: *P* Pointing, *Ps* Pro-social behaviour

^b^NVT, number of valid trials (trials the dog chose a pointed cup); K, dog chose the Knower; G, dog chose the Guesser; – dog did not chose

### Apparatus

**Fig. 1:**
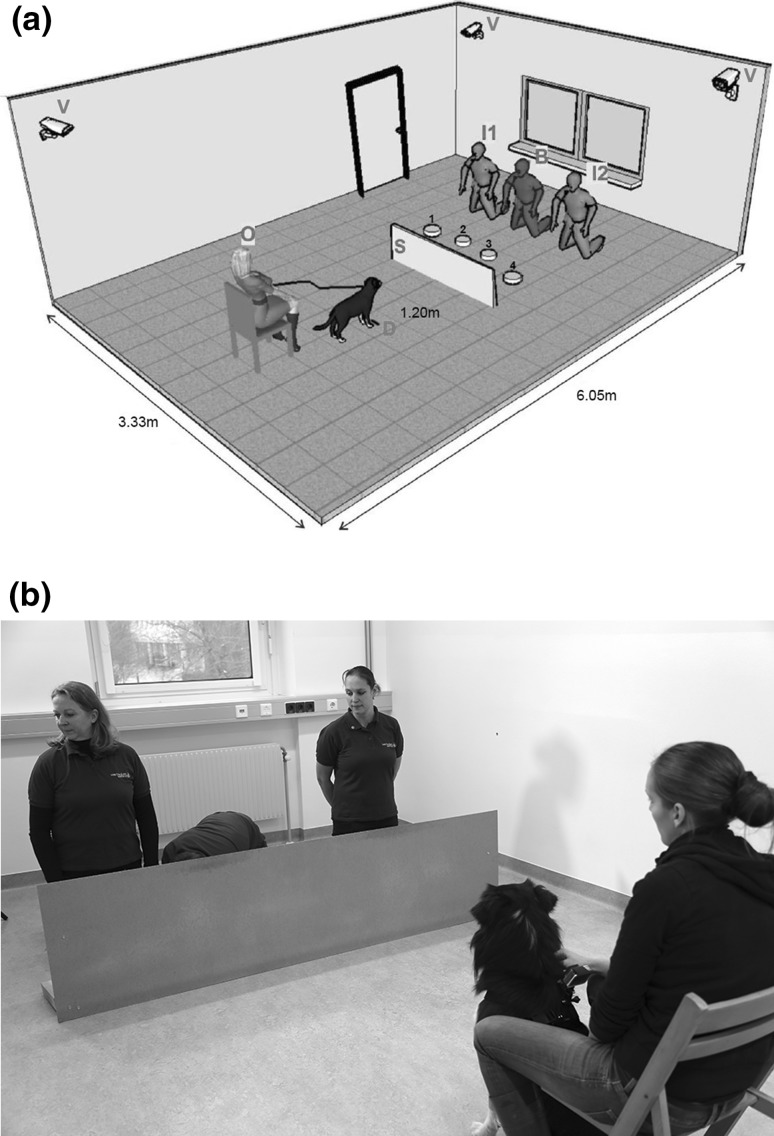
**a.** Sketch of the testing room showing the position of the three video cameras (V), the owner (O), the dog’s releasing point (D), the screen (S), the four containers (1, 2, 3, 4), the two informants (I1 and I2) and the baiter (B) in *blue*, who was only present in GLA condition. **b.** Photograph of informants and baiter (*centre*) in the Guesser Looking Away (GLA) test. Two female experimenters looked down and to the side in identical ways, while the third, male experimenter baited the containers behind the wooden screen and outside of the dog’s and the Guesser’s (left experimenter) but inside the Knower’s (right experimenter) view. Note that the looking side, the identity of the Knower, the position of the Knower, the position of the baited container and the container to be baited were changed pseudo-randomly across trials (see text) (colour figure online)

All tests were conducted in the same 6.05 × 3.33 m large room at the Clever Dog Lab Vienna, which was equipped with a three-camera video recording system (Fig. 1a). The experimental set-up consisted of a removable screen (chipboard; 220 cm × 56 cm), placed at 1.2 m distance from the dog’s release point, and four opaque containers (12.5 cm high × 10 cm in diameter) in a semicircle arrangement, equidistant (1.4 m) from the dog and 45 cm apart from each other. To prevent any noise during baiting, each container was filled with eight layers of paper towel (approximately 2 cm thick in total). The outside of all containers was rubbed with sausage and therefore saturated in smell before each testing session, so that all containers smelled of food regardless of whether they were baited in the respective trial or not. The food used to bait the containers consisted of small pieces of sausage (Frankfurter). The treat supply was kept in an opaque box (15 × 11 × 12 cm), placed behind the experimenters.

#### Procedure

All subjects went through pre-training and three tests of a four-alternative object-choice task following the Guesser–Knower paradigm (Povinelli et al. 1990). Importantly, the pre-training and the first two tests (Guesser Absent and Guesser Present) applied identical procedures as Maginnity and Grace (2014). Each dog completed two sessions separated by approximately 1 week. In the first session, the dogs completed pre-training (ranging from 18 to 23 trials) and, after a 10-min break, either the Guesser Absent or Guesser Present test (24 trials each). In the second session, the dogs completed the remaining two tests (of 24 trials each). The order of presentation was randomized.

#### Pre-training

The goal of the pre-training phase was to accustom the dogs in six consecutive steps to the testing situation and to prevent side or informant preferences. Two informants (AC and BM) were present, but only one at a time hid the treat and subsequently pointed to the baited container. Thus, during pre-training the dog never had to choose between the two informants.

During the first step, just one container was presented to the dog. One of the informants showed the treat (a piece of sausage) to the dog which was sitting centrally in front of the owner, and put the food visibly into the container. After closing the lid, she pointed at the target container with an out-stretched arm and her index finger touching the lid of the container, accompanied by a fixed gaze towards the container. After 2 s, the owner released the dog and it was free to approach the container. When the dog chose the indicated container, the informant opened it and gave the treat to the dog. Then the owner called the dog back to the start position. The identity of the informants and their position (left-/right-hand side of the dog) were pseudo-randomly changed between trials.

In three further steps, the number of containers was increased to four, but only one was baited. In the fifth step, the screen was introduced, which blocked the dog’s view of the baiting process (the hands and the containers). After the silent baiting process, the screen was lowered and the pointing was performed as before. The final step involved, in addition to the screen, the manipulation of all four containers, whereby only one of them was actually baited (but all were rubbed with sausage and smelled of food). The criterion for proceeding to the next step increased from two (steps 1–4) to four and six successful trials in a row.

In the following three tests, the roles of the informants, the baited containers and the pointing positions were counterbalanced and pseudo-randomly determined for each trial prior to the experiment, subject to the constraint that either the Knower or the Guesser did not point to the same container more than twice in a row. Although the owners, like the dogs, could not see the baiting and Clever Hans effects are very unlikely in this context (Hegedüs et al. 2013; Schmidjell et al. 2012), they were instructed to look away from the informants during the baiting and the dog made a choice.

#### Guesser present test

The first test applied the Guesser Present (GP) condition. Two of the four containers were baited (instead of one), and the dogs view was occluded by a screen. The screen was removed and then both informants pointed each to a different, baited container. As both pointers observed the baiting, this test controlled for a preference for a certain informant and any other bias of the dogs. For reasons of consistency with Maginnity and Grace (2014), we called the person who baited the containers the Knower and the other person the Guesser (who actually also observed the baiting in this test).

#### Guesser absent test

The second test applied the Guesser Absent (GA) condition. It tested for the spontaneous discrimination of the two pointers according to their observation of the baiting (Knower vs. Guesser). After the screen was lifted but before the Knower baited one container, the Guesser left the room, returned after the baiting event and, after lowering the screen, pointed to an empty (previously determined) container while the Knower pointed to the correct one.

#### Guesser looking away test

The third, novel test (Guesser Looking Away, GLA) involved a separate baiter in addition to the two informants. Similarly as in Experiment 2 of Maginnity and Grace (2014) the introduction of a third, unfamiliar person controlled for the influence of food handling (both informants now being passive during baiting). As this sudden change in the testing environment had no effect in Maginnity and Grace (2014), we did not consider it having an impact on the dogs’ responses. The baiter knelt between the two informants and, still behind the screen, baited one container. The informants behaved identically during the baiting but had different visual access to the baiter’s actions. The two informants looked down (45° from the horizontal eye line) and in a parallel manner to one, predetermined side (left or right) at an angle of 45° from the line between dog and baiter (Fig. 1b). Importantly, the Knower did not follow the baiter’s hand movement, but looked straight to the side like the Guesser. Therefore, they had differing visual access to the baiter’s actions. Only one (the Knower) could possibly see the baiting, while the other (the Guesser) could not.

#### Analysis

For each trial, we coded which of the four containers the dog chose. A choice was defined as a direct approach towards one container followed by touching or gazing from a close distance (maximally 50 cm) for at least 2 s at this container. If the dog did not make a choice within 60 s, the trial was terminated and recorded as a ‘no response’. If the dog approached a container that was not pointed at, the response was counted as ‘other choice’. Such ‘no responses’ and ‘other choices’ happened only 24 times in all tests of all subjects (1128 total trials) and were excluded from further analyses. For determining Knower preference, we only used choices of pointed containers, i.e., Knower and Guesser choices, with the conservative assumption of chance probability for the Knower’s container being 50%. For each trial, we used the video recordings from three cameras to code which of the four containers the dog chose. Reliability of this coding was verified by a coder who was unfamiliar with the goals of the study, who coded a randomly chosen sample (13%) of video recordings. Due to the high quality of the videos and the ease of determining which container a dog approached, inter-observer agreement was 100% (*κ* = 1).

As in Maginnity and Grace (2014), Knower preference was calculated for each dog and pooled over blocks of 4 trials. We also investigated the occurrence of learning across trials within each test separately by conducting Prism’s linear regression analysis with the average percentage of choice responses made to the Knower in each trial. Knower preferences for individual dogs were assessed with binomial tests. In order to compare the whole sample’s (*N* = 16) performance to chance level, the average preference across dogs for each test was assessed with binomial tests. In order to compare the performance of the dogs in the current study with those of Maginnity and Grace (2014), an independent samples *t* test was performed for the GA, GP, and GLA tests.

#### Ethical note

This study was approved in accordance with good scientific practice guidelines and national legislation by the Ethical Committee of the University of Veterinary Medicine Vienna (Ref: ETK-10/02/2016). All experimental procedures were performed in compliance with the Austrian Federal Act on the Protection of Animals (Animal Protection Act–TSchG, BGBl. I Nr.118/2004). All tests were completely non-invasive and therefore, according to the Austrian Animal Experiments Act (§ 2, Federal Law Gazette No. 501/1989), are not considered as animal experiments and do not require obtaining special permission. All dog owners gave written consent to participate in the study.

## Results

Overall dogs responded to the location pointed at by the Knower or the Guesser on 97.9% of trials (*n* = 1128). As in Maginnity and Grace (2014), trials with no response (*N* = 5) or in which the dog chose a container that was not pointed at occurred rarely (*N* = 19), and were omitted from subsequent analyses.

**Fig. 2:**
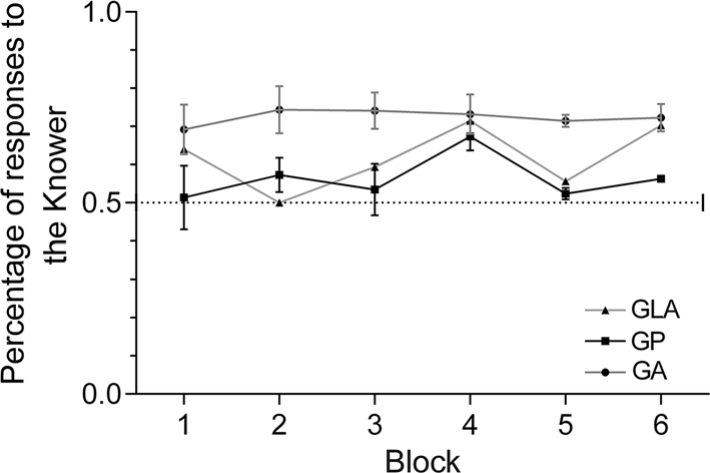
Average percentage of choice responses made to the Knower per block of four trials in the three tests (GP, GA and GLA). The dashed line indicates chance responding. *Bars* indicate one standard error (SE)

Figure 2 shows the percentage of choice responses made for the Knower across successive blocks of four trials for all three tests. On a group level, Knower preference was significantly greater than chance (50%) in two tests (GA: mean = 72.3%, *t*(15) = 7.46, *p* < 0.0001; GLA: mean = 61.7%, *t*(15) = 4.89, *p* = 0.0002) and approached significance in one (GP: mean = 56.2%, *t*(15) = 1.99, *p* = 0.0643). When the data were pooled over trials in which the informants had differential knowledge of food location (GA and GLA), providing a more sensitive test of the consistency of individual differences (Maginnity and Grace 2014), the average Knower preference was 67% (95% CI 62–71%), and ranged from 49% (an 8.5-year-old male Mixed Spitz named Patrasch) to 85% (a 1.5-year-old female Mix named Clio).

We also assessed the performances of individual dogs in the three tests (Table 1). Each dog’s performance was tested against chance using a binomial test (*p* < 0.05). While in the GP test only one dog (Cameron) showed a significant Knower preference, eight dogs in the GA test and three dogs in the GLA test showed this preference. Only one dog (Bucksi) showed a Guesser preference, this was in the GP test. Importantly, even if the three individually significant dogs in the GLA test were excluded from the analysis, the group performance remained significant (mean = 58.7%, *t*(12) = 4.316, *p* < 0.01). Concerning the first-trial performance, 62.5% of dogs (10/16) chose the Knower in the GP condition (*p* = .23, binomial test), 73.3% (11/15) in the GA condition (*p* = 0.06), and 81.3% (13/16) in the GLA condition (*p* = .01). Table 1 shows the first-trial performance of each dog in each test.

**Fig. 3:**
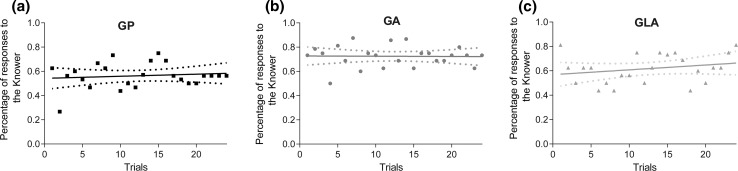
Average percentage of choice responses made to the Knower in each trial of the three tests (GP, GA and GLA) and lines fitted to the data using Prism’s linear regression analysis. The *dashed lines* show the 95% confidence interval

A possible effect of learning within the three 24-trial tests was tested by examining changes in the dogs’ performance across trials. Prism’s linear regression analysis revealed no effect of learning in any of the tests [GP: *F*(1,22) = 0.302, *p* > 0.58; GA: *F*(1,22) = 0.006, *p* > 0.93, GLA: *F*(1,22) = 1.293, *p* > 0.26; Fig. 3]. Furthermore, the average Knower preferences of the first 4-trial block in each test did not differ from the average of the whole 24-trial test (*t* tests for GP, GA and GLA, *p* = 0.4125, 0.5180 and 0.6828, respectively).

**Fig. 4:**
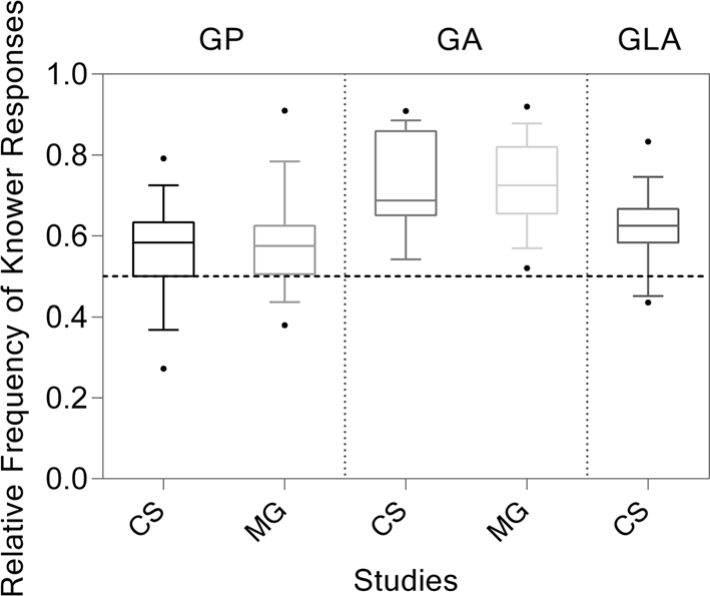
Relative frequencies of Knower responses in the Guesser Absent and Guesser Present tests of Experiment 1 of Maginnity and Grace (2014) (MG) and in the three tests of the current study (CS). The bottom, middle and top horizontal lines of each box show the 25th, 50th (median) and 75th percentiles, respectively. The whiskers extend to the 90th percentile

The results from the independent samples *t* tests revealed no significant differences in the percentage of Knower choices between the dogs of both studies, Maginnity and Grace (2014) and the present one, in the two identical tests (GP: mean 58 vs. 56%, respectively; *p* = 0.706, GA: mean 73 vs 72%, respectively, *p* = 0.9; Fig. 4).

## Discussion

The current study replicated the results of Maginnity and Grace (2014) and therefore proved the robustness of the findings, but additionally added a crucial novel test. Similarly to the previous study, dogs chose the help of a Knower (here the baiter) but ignored the help of a Guesser (who left the room during the baiting) in the GA test. Like in the original study with chimpanzees (Povinelli et al. 1990) and an earlier study with dogs (Cooper et al. 2003), the subjects showed a spontaneous and therefore unlearned ability to use cues related to the informants’ presence during the baiting. The absence of a significant informer preference in the GP test, in which one informant performed the baiting, but both watched the baiting, excludes the possibility that the Knower preference in GA arose from a baiter preference. It is also unlikely that it arose from different pointing behaviour of the two experimenters, as they alternated roles within a test session and were both equally informed about the food location. The fact that in the GP test the dogs showed a tendency to follow the person who baited the box suggests an influence of cues related to food handling, but cues correlated with the informants’ attentional state (i.e., whether the informants had attended the food baiting; here both informants) seem to have prevented a stronger, significant preference for the baiter. A further control with the Guesser handling the food would be necessary to determine whether it is the attentional state or the food handling that guided this behaviour.

The two informants in the GA test, like in most Guesser–Knower tasks in previous studies, differed not only in what they saw, leading to different knowledge states, but also in the way they behaved (remaining in the room or leaving it) during the hiding process. In Experiment 3 of Maginnity and Grace (2014), they showed clearly discriminable behaviour (watching the baiting versus looking at the ceiling). This offers two possibilities for attention cues that are used by the dogs to decide whom to follow. Since there is evidence that dogs are sensitive to cues relating to human attention (Agnetta et al. 2000; Hare and Tomasello 1999; McKinley and Sambrook 2000; Schwab and Huber 2006; Soproni et al. 2002), and can use a glancing cue to locate food (Miklósi et al. 1998), the dogs may avoid or ignore people because they are not attentive.

The alternative to attentional cues is behavioural cues. Dogs may avoid people who are looking at the ceiling, a behaviour that dogs usually do not see in everyday life conditions in association with food provisioning, and for which they therefore have no associations. Pre-experimental learning about human behaviour and its consequences may fully account for the results in the GA test (Gagliardi et al. 1995; Roberts and Macpherson 2011; Udell et al. 2011; Viranyi and Range 2011).

The GLA test in the present study controls for the possibility that the dogs use obvious, i.e., directly observable behavioural cues, because both informants showed identical looking behaviour. However, due to their different positions in the room only one of the informants could see the baiting. Note that during baiting the dogs were not able to see the containers behind the screen, and therefore, they could not utilize the eye-object line of the Knower. Only by inferring who could see the baiting, could dogs establish a preference for the container indicated by the Knower.

It is noteworthy that from the tested sample of 16 dogs only three showed a significant preference for the Knower. Overall there was quite a high degree of variation between individuals but also between tests and between blocks of trials. Given that a sudden distraction, a slight drop in attentiveness due to creeping fatigue would easily cause failures, this variation may not be very surprising. Nevertheless, even if the three individually successful dogs were excluded from the analysis, the group’s preference for the Knower remained significant in the GLA test, confirming the robustness of the results. It is furthermore noteworthy that the significant performance in the GLA test was not the result of a learning process, because 13 of 16 subjects chose the Knower in the first trial and this level of Knower preference did not change in the course of test.

Together, these results provide evidence that dogs discriminate between two possible informants on the basis of subtle perceptual cues, their lines of sight. The dogs tracked the informants’ gaze direction geometrically (Tomasello et al. 1999), thereby appreciating the difference between their own and the informant’s line of sight. Although gaze following seems to be elicited in a reflexive manner and exists in many vertebrates (review in Fitch et al. 2010), including solitary reptiles (Wilkinson et al. 2010), the following of others’ gaze direction into distant space or around barriers seems to be a less common and more flexible mechanism (Wallis et al. 2015). In the popular barrier test, the observers need to reposition themselves, either physically or mentally, to check or imagine the others’ target of attention. This may be achieved by either representing the looker’s visual perspective (Povinelli and Eddy 1996b) or by learning how visual barriers impair perceptions (Tomasello et al. 1999).

Unsurprisingly, we see no or little evidence in many non-human species to use the gaze direction of a human experimenter, or a conspecific, as a cue to find hidden food (Anderson et al. 1996; Call et al. 2000; Schloegl et al. 2008). Great apes (Bräuer et al. 2005; Rosati and Hare 2009) and two corvid species, ravens and rooks (Bugnyar et al. 2004; Schloegl et al. 2008), have proven this ability first, canines followed later. Wolves not only performed well at using conspecifics’ cues, but hand-raised and thus properly socialized subjects also exploited human gaze cues to track gaze behind barriers (Range and Virányi 2011). Dogs have also proven capable of following the gaze of humans into distant space (Wallis et al. 2015) and around a barrier in a food searching context (Met et al. 2014).

Although geometrical gaze following is thought to rest on a cognitively sophisticated mechanism (Fitch et al. 2010), it does not require mind-reading; the recognition of mental states like beliefs, desires, and intentions. The dogs’ confidence in the informant who was in the position to see the relevant event (food hiding) might possibly emerge from an awareness of the superior knowledge state of the Knower over the Guesser, but a more parsimonious explanation of this behaviour is in terms of generalization from similar situations in everyday life (Udell et al. 2011). Pet dogs may have experienced reinforcement in similar, but not identical situations. They can experience on a daily basis that it is easier to communicate with humans whose eyes are visible, in contrast to humans whose eyes are covered (Viranyi and Range 2011) and with humans who look at instead of above a target (Soproni et al. 2001). Similarly, they can learn about the consequences of humans looking towards instead of away from objects. By using geometrical gaze following these experiences could be generalized to temporarily invisible objects and might have led to the reluctance of dogs to follow the looking-away person in the GLA test.

Altogether, the findings of the present study provide evidence that canines are able to react to what others can or cannot see. This ability seems to be based on the use of occurrent gaze cues in a cooperative situation and is thus at a lower level of representation than a full-blown competence of attributing the concept ‘seeing’ (Buckner 2014). Scrub jays acting on the basis of a memory of past gaze cues (Dally et al. 2006) and chimpanzees acting on the basis of remembered preferences of others (Schmelz et al. 2011) are one step closer to a genuine representation of ‘seeing’. Even more exciting is the recent demonstration that common ravens take into account the visual access of others, even when they cannot see, but can hear, a conspecific (Bugnyar et al. 2016). Rather than tracking correlations between head cues and a competitor’s behaviour, they seem to infer what others can or cannot see and therefore do or do not know.

It remains thus an open question whether canines are also able to develop and attribute a cue-less concept of ‘seeing’. In contrast to the findings in chimpanzees and corvids, dogs represent a model of non-human perspective taking in a cooperative and hetero-specific context. So far, dogs have been found to be excellent behaviour-readers, highly competent in learning about directly observable behavioural, gestural, vocal, and attentional cues. The present findings provide further evidence to the dog’s ability to act on the basis of the human’s visual access to the food (Kaminski et al. 2009; Kaminski et al. 2013). Like wolves (Range and Virányi 2011), they are able to reposition themselves to follow a gaze cue when faced with a barrier blocking their view. Insofar as can be ascertained, it is an open question to what degree phylogenetic (‘domestication’) and ontogenetic (individual experience) influences gave rise to this important element of Theory of Mind (Udell et al. 2010). Still nevertheless, this capacity is of high adaptive value for life in the human environment.
